# β-Carboline Alkaloids in Soy Sauce and Inhibition of Monoamine Oxidase (MAO)

**DOI:** 10.3390/molecules28062723

**Published:** 2023-03-17

**Authors:** Tomás Herraiz

**Affiliations:** Instituto de Ciencia y Tecnología de Alimentos y Nutrición (ICTAN), Spanish National Research Council (CSIC), José Antonio Nováis 6, Ciudad Universitaria, 28040 Madrid, Spain; therraiz@ictan.csic.es

**Keywords:** monoamine oxidase, MAO inhibition, soy sauce, β-carbolines, norharman, harman, tetrahydro-β-carbolines, alkaloids, heterocyclic amines

## Abstract

Monoamine oxidase (MAO) oxidizes neurotransmitters and xenobiotic amines, including vasopressor and neurotoxic amines such as the MPTP neurotoxin. Its inhibitors are useful as antidepressants and neuroprotectants. This work shows that diluted soy sauce (1/3) and soy sauce extracts inhibited human MAO-A and -B isozymes in vitro, which were measured with a chromatographic assay to avoid interferences, and it suggests the presence of MAO inhibitors. Chromatographic and spectrometric studies showed the occurrence of the β-carboline alkaloids harman and norharman in soy sauce extracts inhibiting MAO-A. Harman was isolated from soy sauce, and it was a potent and competitive inhibitor of MAO-A (0.4 µM, 44 % inhibition). The concentrations of harman and norharman were determined in commercial soy sauces, reaching 243 and 52 μg/L, respectively. Subsequently, the alkaloids 1,2,3,4-tetrahydro-β-carboline-3-carboxylic acid (THCA) and 1-methyl-1,2,3,4-tetrahydro-β-carboline-3-carboxylic acid (MTCA) were identified and analyzed in soy sauces reaching concentrations of 69 and 448 mg/L, respectively. The results show that MTCA was a precursor of harman under oxidative and heating conditions, and soy sauces increased the amount of harman under those conditions. This work shows that soy sauce contains bioactive β-carbolines and constitutes a dietary source of MAO-A and -B inhibitors.

## 1. Introduction

Monoamine oxidase (MAO) is an enzyme located at the outer membranes of mitochondria in the brain, liver, intestinal mucosa, and other organs that catalyzes the oxidative deamination of biogenic amines, neurotransmitters, vasoactive dietary amines and xenobiotic amines, including dopamine, serotonin, norepinephrine, tyramine, tryptamine and the MPTP (1-methyl-4-phenyl-1,2,3,6-tetrahydropyridine) neurotoxin. MAO appears as two isozymes, MAO-A and B, which are distinguished by substrate and inhibitor selectivity [1]. MAO-A preferentially catalyzes the oxidation of serotonin and norepinephrine and is inhibited by clorgyline, whereas MAO-B selectively catalyzes the oxidation of phenylethylamine and benzylamine and is inhibited by (R)-deprenyl. Tyramine, dopamine, and tryptamine are substrates for both enzymes. MAO isozymes play a significant role in the metabolism and regulation of neurotransmitters and biogenic amines and are involved in several diseases [1,2]. Thus, MAO-B has been implicated in neurological disorders and diseases and MAO-A in psychiatric conditions and depression [3]. An increased number of inhibitors of MAO, both of synthetic and natural product origin, are currently a matter of interest in drug discovery [1,4], both as antidepressants (MAO-A inhibitors) [3,5] or neuroprotectants (MAO-B inhibitors) [6,7,8,9,10]. The oxidation of biogenic amines and neurotransmitters by MAO produces hydrogen peroxide (H_2_O_2_), ammonia and aldehydes, which represent risk factors for oxidative cell injury and toxicity [11,12]. MAO also bioactivates proneurotoxins such as MPTP [13,14,15]. The inhibition of MAO could reduce reactive oxygen species or toxins [16,17]. On the other hand, the inhibition of peripheral MAO (e.g., intestinal and liver) has been linked to hypertensive crisis produced by a reduced metabolism of vasopressor dietary amines (e.g., tyramine) [1,18]. In this regard, the inhibition of MAO may potentiate the physiological effects of dietary vasoactive amines and exert possible food-drug interactions. The available inhibitors are sometimes irreversible inhibitors that may produce hypertensive crises, and in this regard, new inhibitors devoid of those undesirable effects are currently needed.

Dietary and environmental factors such as smoking, foods, herbal preparations or drugs might affect the action of MAO. MAO is reduced in smokers compared to nonsmokers [19,20], and cigarette smoke inhibits MAO-A and -B isozymes [21]. Naturally occurring β-carboline alkaloids have been identified in the inhibition of MAO by cigarette smoke [21,22], coffee [23] and raisins [24]. The inhibition of MAO in smokers and coffee drinkers might be linked with some biological actions, such as the addictive properties of cigarettes and depression [25,26] and the lowest incidence of Parkinson’s disease (PD) [27,28,29,30]. More research is currently needed to study the effects of diet and environmental factors on MAO enzymes. On the other hand, new inhibitors of MAO devoid of undesirable effects might arise from naturally occurring compounds and sources. β-Carbolines are naturally occurring bioactive alkaloids that exhibit an array of pharmacological effects such as the binding to benzodiazepine, imidazoline, serotonin and opiate receptors as well as inhibition of kinases and MAO [14,31,32,33,34,35,36,37,38,39]. Some of these substances are also involved in toxicological effects [14,15]. β-Carbolines can be absorbed and accumulated in tissues contributing to their presence and actions in the biological systems [40,41,42]. These alkaloids have been found in foods and seasonings [22,24,43,44,45,46,47,48]. In this regard, soy sauce is a special seasoning made from salt, water, soybeans and wheat that, after heating processes, is traditionally fermented in a process involving molds, lactic bacteria and yeast lasting months, and this process may result in an increase of β-carboline alkaloids [49]. This research aimed to study the presence of β-carboline alkaloids in soy sauce and to assess the activity and inhibition of MAO by soy sauce using a chromatographic assay. It is shown that MAO isozymes are inhibited in vitro by soy sauce and soy sauce extracts, suggesting the presence of inhibitors. The aromatic β-carboline alkaloids norharman and harman were identified and analyzed in soy sauce, and harman was subsequently isolated as a potent and competitive inhibitor of MAO-A. Finally, tetrahydro-β-carboline-3-carboxylic acid (THβC-3-COOH) alkaloids were analyzed in soy sauce, and it was found that 1-methyl-1,2,3,4-tetrahydro-β-carboline-3-carboxylic acid (MTCA) was the precursor of the MAO inhibitor harman. 

## 2. Results

**Figure 1:**
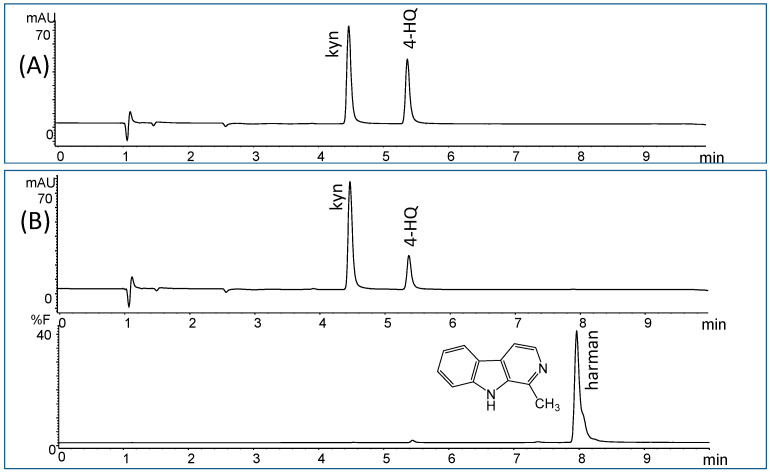
HPLC analysis of MAO enzymatic assays. Control assay (**A**) and the assay in the presence of the inhibitor harman isolated from soy sauce (**B**). The product of the deamination of kynuramine (kyn) is 4-hydroxyquinoline (4-HQ).

**Figure 2:**
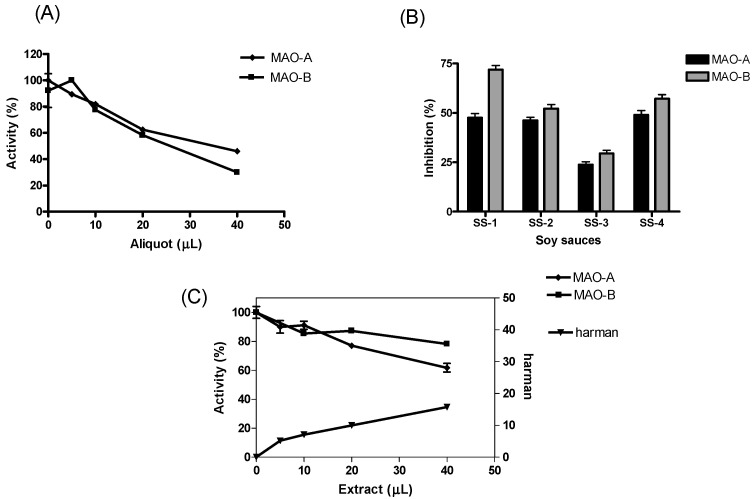
(**A**) Inhibition of MAO-A and -B in the presence of increased aliquots of soy sauce (SS-4) diluted 1/3 in buffer-10% DMSO. (**B**) Inhibition (%) of MAO-A and -B by different commercial soy sauces (30 μL of soy sauce diluted 1/3 in buffer-10% DMSO). (**C**) Inhibition of MAO by extract of PRS (buffer phosphate-MeOH 1:1) and increased presence of harman (fluorescence area). Results are mean ± SEM.

The enzymatic activity of human MAO-A and -B was assayed by chromatographic means following HPLC analysis of 4-hydroxyquinoline (4-HQ) formed by enzymatic deamination of kynuramine (kyn) substrate (Figure 1A). This chromatographic analysis allows the determination of the inhibition of MAO without interferences that may occur when using inhibitors present in complex mixtures [17,50]. The activity of MAO was studied in the presence of increasing concentrations of soy sauce. Both MAO-A and -B isozymes were inhibited in the presence of soy sauce (Figure 2A). This inhibition was reversible for MAO-A (90%) and partly reversible for MAO-B (60%), as suggested from the activity recovered following incubation of MAO with soy sauce when compared with controls. The inhibition of MAO-A and -B obtained with different samples of soy sauces (1/3 diluted) ranged from 23–50% for MAO-A and 30–73% for MAO-B (Figure 2B). In the assays, the standard inhibitors of MAO, clorgyline (1 µM) and *R*-deprenyl (1 µM) provided more than 90% inhibition of MAO-A and -B, respectively. 

**Figure 3:**
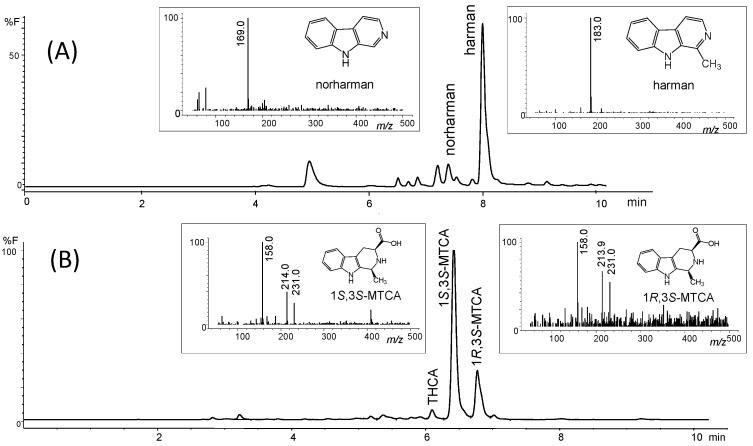
(**A**) Chromatogram of the aromatic β-carbolines isolated from soy sauce by HPLC-fluorescence (300 nm exc./433 nm emiss.), and mass spectra of the compounds identified by HPLC-MS analysis. (**B**) Chromatogram of THβC-3-COOH from soy sauce by HPLC-fluorescence (270 nm exc./343 nm emiss.) and mass spectra of MTCA isomers obtained by HPLC-MS analysis (Fragmentor: 100 V).

**Figure 4:**
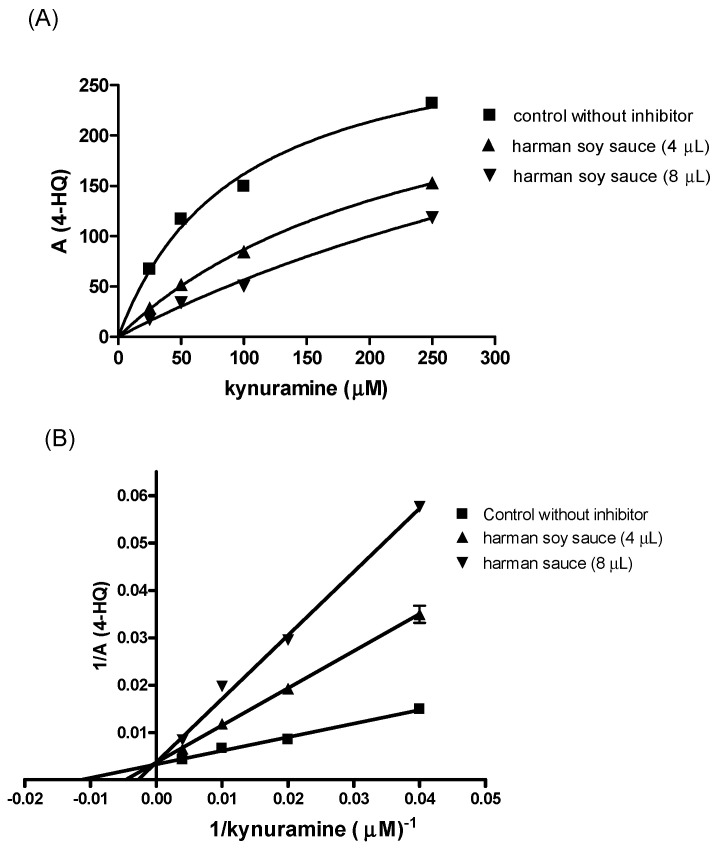
(**A**) Curves of inhibition of MAO-A by harman isolated from soy sauce in the presence of increasing concentrations of kynuramine. (**B**) Lineweaver-Burk kinetic plots of the inhibition of harman isolated from soy sauce. The calculated concentration of harman for 8 μL in the assay was 0.4 μM.

The inhibition of the deamination of kynuramine by MAO suggested the presence of inhibitors in soy sauces. Then, soy sauce with a relatively high inhibition of MAO was fractionated by solid phase extraction (SPE) using propylsulfonic (PRS) columns. The isolated extract inhibited MAO-A and, to a lesser extent, MAO-B (Figure 2C), suggesting that it contained compounds contributing to the inhibition of MAO-A. When considering dilutions, a similar degree of inhibition of MAO-A was observed with both soy sauce and their PRS extracts, suggesting that PRS extracts contained compounds that were contributors to MAO-A inhibition in soy sauce. The analysis of those PRS-extracts evidenced the presence of two β-carbolines that were identified by co-injection with standards, UV-VIS spectra and HPLC-MS as norharman (*m*/*z* at 169 (M + H)^+^) and harman (*m*/*z* at 183 (M + H)^+^; Figure 3A). Harman that was the major β-carboline in the PRS extracts, was isolated following successive RP-HPLC chromatographic injections by collecting the chromatographic peak at the end of the Diode Array Detector (DAD) and then used to study inhibition (Figure 1B and Figure 4A). This β-carboline from soy sauce was a potent inhibitor of MAO-A and showed a competitive type of inhibition when determined at different concentrations of substrate and inhibitor (Figure 4B). The inhibition of MAO-A by the harman isolated from soy sauce was in agreement with the concentration of harman included in the assays and its IC_50_ [21]. Thus, harman from soy sauce (0.4 μM) gave a 44 % inhibition of MAO-A using kynuramine (250 μM).

**Figure 5:**
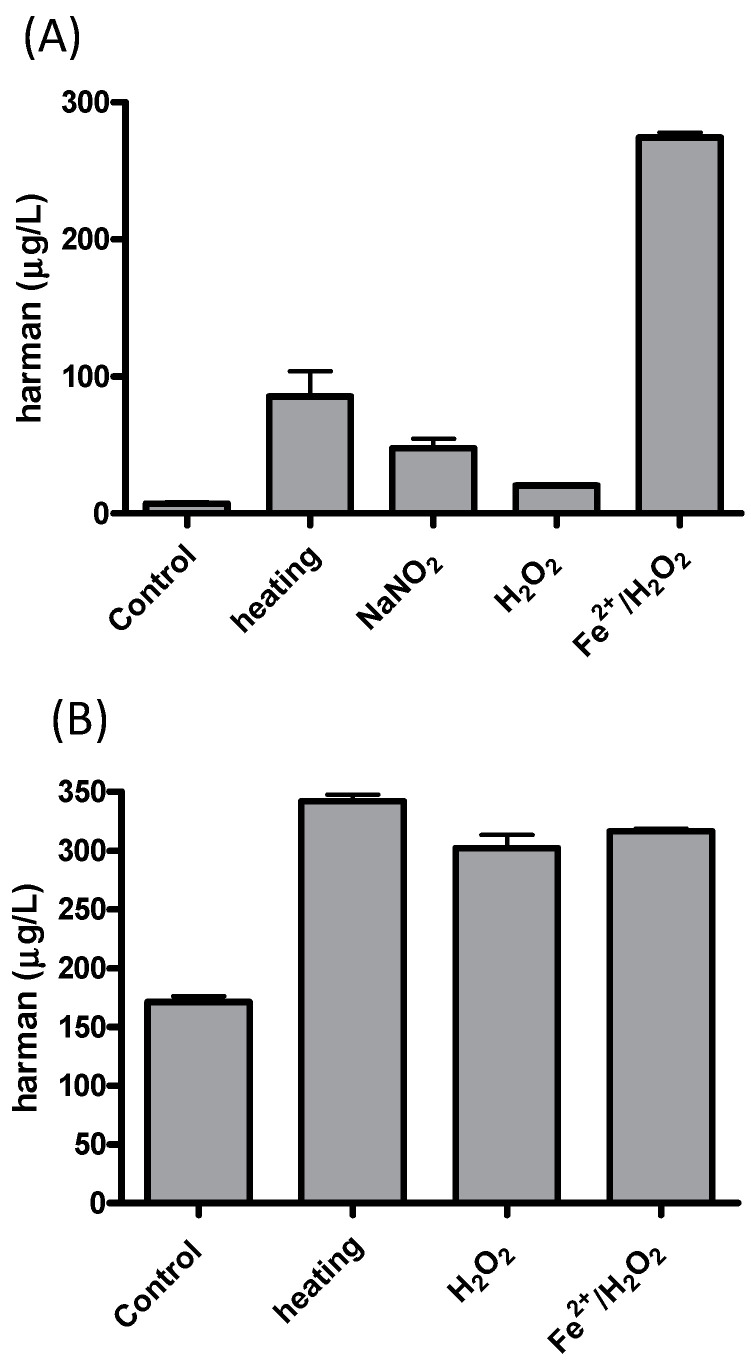
Formation of harman from MTCA (50 μM) in phosphate buffer, pH 4 (**A**) and in soy sauces under several conditions of heating and oxidation (**B**) (see experimental section).

**Figure 6:**
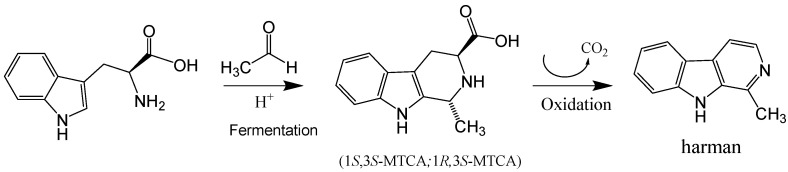
Formation of MTCA during fermentation of soy sauce through Pictet-Spengler condensation of tryptophan with acetaldehyde and its subsequent oxidation and decarboxylation to give harman, an inhibitor of MAO-A, identified in soy sauce.

The aromatic β-carboline alkaloids norharman and harman were measured in commercial samples of soy sauces by SPE and HPLC-fluorescence detection (Table 1). Harman was a major aromatic β-carboline, and its concentration varied among different samples of soy sauces ranging from 46.7 to 243 μg/L, whereas norharman ranged from 11.6 to 52 μg/L. Subsequently, soy sauces were analyzed for the presence of tetrahydro-β-carboline-3-carboxylic acids (THβC-3-COOH) that are precursors of the aromatic β-carboline alkaloids [44,51,52,53]. Soy sauces contained 1,2,3,4-tetrahydro-β-carboline-3-carboxylic acid (THCA) and 1-methyl-1,2,3,4-tetrahydro-β-carboline-3-carboxylic acid (MTCA; Figure 3B) that were identified by chromatographic and mass spectrometric analysis (i.e., *m*/*z* at 217 (M + H)^+^ for THCA, and *m/z* at 231 (M + H)^+^ for MTCA). THβC-3-COOHs were determined following SPE, and their concentration was up to 1000 times higher than that of aromatic βCs (Table 1). The main THβC-3-COOH was MTCA which appeared as two diastereoisomers (1*S*,3*S* and 1*R*,3*S*) with the same spectra (Figure 3B) and concentration reached 450 mg/L. The compound MTCA was isolated from soy sauce by successive HPLC injections, and it afforded harman when heated (90 °C, 6 h; results not shown), showing that it is a direct precursor of this aromatic β-carboline. The formation of harman from MTCA was studied, and it afforded harman when treated under conditions of heating and oxidation (Figure 5A). Moreover, a sample of soy sauce increased the level of harman under conditions of heating and oxidation (Figure 5B). Therefore, these results indicate that MTCA was the precursor of harman, a potent inhibitor of MAO-A, in soy sauce (Figure 6).

## 3. Discussion

The results of this work have shown that MAO isozymes (MAO-A and -B) are inhibited in vitro by soy sauce aliquots and soy sauce extracts suggesting the presence of inhibitors in soy sauce. They also have shown the occurrence of aromatic β-carboline alkaloids in soy sauce extracts inhibiting MAO. The β-carbolines, norharman and harman, were identified and analyzed, and among them, harman was a major compound. Subsequently, harman was isolated from soy sauce, and it was a potent inhibitor of MAO-A. The level of β-carboline alkaloids highly varied among the samples of soy sauces analyzed (46 to 243 μg/L for harman). The amount of β-carbolines in soy sauce is relatively high when compared with other foodstuffs [22]. Besides aromatic β-carboline alkaloids, the results in this work also show the presence of the tetrahydro-β-carboline-3-carboxylic acids (THβC-3-COOHs) THCA and MTCA in soy sauce. THβC-3-COOHs in foods form through the reaction of tryptophan with formaldehyde (THCA) or acetaldehyde (MTCA) [45]. Thus, MTCA forms during fermentation and storage as tryptophan reacts with acetaldehyde released (Figure 6) [45,49,54,55]. Soy sauce is made from a mixture of salt, water, soybeans and wheat that, after heating processes, is fermented in a process successively involving molds (*Aspergillus oryzae*), lactic bacteria and yeast that may last months, and that is finally filtered and heat-treated for sterilization. Alternatively, elaboration methods may involve the chemical hydrolysis of soybeans or chemical hydrolysis and fermentation. Then, factors such as the fermentation process, time of storage and heating processes could determine the content of THβC-3-COOH. For instance, soy sauces contain a high level of THβC-3-COOHs [45], and MTCA appeared up to 1000 times higher in concentration than harman (Table 1). Results here have shown that MTCA is the precursor of harman in soy sauce (Figure 5 and Figure 6). Harman increased in soy sauce under heating and oxidative conditions. Therefore, technological processes such as high temperatures and long storage time or oxidation could increase the levels of the MAO inhibitor harman in soy sauce. These results agree with previous results on the formation of β-carbolines from THβC-3-COOHs [44,51,53,56]. Previously, MTCA received attention as a possible precursor of mutagens when reacted with nitrite [57]. The reaction of MTCA with nitrite affords harman among other compounds, as proved during nitrosation of THβCs [56].

β-Carbolines are pyrido-indole alkaloids that occur and accumulate in biological tissues [31,34,41,42,58,59,60,61,62]. β-Carbolines also occur in foods and tobacco smoke, suggesting daily exposure to these compounds [22,44,46]. It is assumed that their occurrence in foods such as soy sauce, along with their absorption, may contribute to their physiological occurrence and accumulation in the body. Remarkably, β-carbolines exert various pharmacological and psychopharmacological effects, including antidepressant-like effects [32,37,38,61,63]. They alter the concentrations of brain neurotransmitters by interaction with serotonin, benzodiazepine, opioid and imidazoline receptors and also interact with MAO, kinases and cytochrome P450 enzymes [14,31,32,33,34,35,39,64,65]. The presence of these compounds in the human brain has been involved in alcoholism and addiction and they have been investigated as potential endogenous and/or environmental proneurotoxins involved in Parkinson’s disease [13,14,15,59,66,67]. The latter is based on the fact that under bioactivation by N-methyltransferases, β-carbolines afford neurotoxic N-methyl-β-carbolinium cations structurally resembling the neurotoxin MPP^+^ that is produced from MPTP with the participation of MAO [62,67]. Moreover, endogenous β-carbolines might somehow affect the metabolism of exogenous amines and neuroamines, exerting potential biological actions [23,24,61]. 

Further studies are needed to fully delineate the implications of dietary, environmental, and endogenous β-carboline alkaloids in human health. Some of the effects attributed to β-carboline alkaloids could be produced by their interaction with MAO enzymes [21,23,68]. The primary role of MAO isozymes in the CNS lies in the metabolism of amines and the regulation of neurotransmitter levels and intracellular amine stores. In the gastrointestinal tract, the circulatory system and the liver, MAO regulates the levels of exogenous dietary amines that exert vasopressor effects serving a protective function. The use of MAO inhibitors, particularly irreversibly inhibitors, may cause a hypertensive crisis when the patients consume tyramine-containing foods (the so-called “cheese effect”) [1,18,69,70]. MAO also metabolizes toxic xenobiotic amines such as the neurotoxin MPTP and, in this regard, the inhibitors of MAO can be protective agents [13,14,71,72]. Moreover, the oxidation of amine substrates by MAO results in the production of hydrogen peroxide, ammonia, and aldehydes, which are risk factors for cell oxidative injury [12,17,73]. Therefore, the use of MAO-inhibiting substances can protect against toxicants and oxidative stress [11,17,71,74]. Currently, MAO inhibitors are being developed against Parkinsonism and neurodegeneration and as antidepressant drugs [1,3,4,5,6,7,8,9]. Inhibition of MAO could help to spare neurotransmitters such as dopamine, serotonin and others while reducing reactive oxygen species. Alternatively, the inhibition of peripheral MAO could eventually affect the metabolism of exogenous and dietary amines like tyramine or tryptamine, potentiating vasopressor effects [1,3]. This work has shown the presence of β-carboline alkaloids in soy sauce, which are inhibitors of MAO. Based on concentration, other substances can contribute to MAO inhibition in soy sauce in addition to β-carbolines. However, β-carbolines could be absorbed and accumulated in tissues exhibiting MAO inhibition [62]. Soy sauce products might be beneficial against neurodegenerative diseases [75,76]. This study shows that soy sauce contains MAO inhibitors, and the inhibition of MAO is a target in neuroprotection. 

## 4. Materials and Methods

Kynuramine, 4-hydroxyquinoline, norharman (9*H*-pyrido-(3,4-b)-indole), harman (1-methyl-9*H-*pyrido-(3,4-b)-indole) and 1,2,3,4-tetrahydro-β-carboline-3-carboxilic acid (THCA) and 1-methyl-1,2,3,4-tetrahydro-β-carboline-3-carboxilic acid (1*S*,3S-MTCA) were purchased from Sigma. Recombinant human monoamine oxidase A and B were obtained from Gentest. HPLC grade acetonitrile, methanol and dimethyl sulfoxide (DMSO) were from Scharlau, Barcelona (Spain), and dichloromethane from Merck, Darmstad (Germany). Commercial samples of soy sauces from different producers and origins, also labeled as produced from natural fermentation, were purchased in local supermarkets. Sample preparation of soy sauces for analysis and enzyme inhibition was carried out in several ways: (a) diluted soy sauces (1/3) with phosphate buffer pH 7.4–10% DMSO were used for MAO inhibition; (b) soy sauces were fractionated by SPE, and the eluting fraction of TP-MeOH used for analysis and MAO inhibition; and (c) the β-carboline harman was isolated by HPLC and subsequently used for MAO inhibition and kinetic studies.

### 4.1. Isolation of Tetrahydro-β-carboline-3-carboxylic Acid and β-Carboline Alkaloids from Soy Sauces

(a) Tetrahydro-β-carboline-3-carboxylic acids (THβC-3-COOHs) were analyzed from soy sauces by using benzenesulfonic acid (SCX)-columns following a previously described procedure that affords good performance and reliability with recoveries higher than 90% [45,54]. The elution fraction of 0.4 M phosphate buffer-methanol (1:1), pH 9, containing the THβC-3-COOH was injected into RP-HPLC-fluorescence (excitation, 270 nm; emission, 343 nm) for quantitative analysis and into HPLC-MS for identification, as mentioned below. Quantitation was carried out from calibration curves constructed with standards of THβC-3-COOH isolated under the same procedure [45,54].

(b) The aromatic β-carbolines were isolated for subsequent chromatographic analysis, identification and MAO inhibition studies by using a solid phase extraction procedure reported before that affords good performance and reliability with recoveries higher than 90% [22,46]. Briefly, soy sauce diluted with 0.6 M HClO_4_ and added with 125 μL 1-ethyl-β-carboline (0.2 mg/L) as internal standard was passed through a propylsulfonic acid (PRS)-derivatized silica column that was eluted with water (6 mL), 0.4 M phosphate buffer, pH 9 (3 mL) and 0.2 M buffer phosphate-methanol (1:1), pH 9 (3 mL). The eluates of 0.2 M buffer phosphate-methanol (1:1), pH 9 (3 mL) containing the β-carbolines, were analyzed by HPLC and norharman and harman detected by fluorescence (300 nm, excitation and 433 nm, emission). Quantitation was obtained from calibration curves of standards isolated under the same procedure. The same PRS-SPE procedure but without an internal standard was used to isolate the fractions containing β-carboline for subsequent MAO inhibition studies. In this case, the eluting fractions of 0.4 M phosphate buffer, pH 9 (3 mL) and 0.2 M buffer phosphate-methanol (1:1), pH 9 (3 mL) were mixed and used for MAO inhibition. In order to isolate the β-carboline harman, PRS fractions of 0.2 M buffer phosphate-methanol (1:1) pH 9 were evaporated under vacuum and extracted with dichloromethane (Merck). The organic phase was evaporated, redissolved and injected into HPLC as mentioned below, with the peak corresponding to the β-carboline harman collected from successive HPLC injections. After removing acetonitrile, harman was extracted with dichloromethane in pH 9, concentrated to dryness, and redissolved in phosphate buffer containing 30% DMSO, and used for inhibition of MAO. Corresponding blanks following the same procedures but without β-carbolines were used in the assays.

### 4.2. Formation of Harman from 1-Methyl-1,2,3,4-Tetrahydro-β-Carboline-3-Carboxylic Acid (MTCA)

To study the formation of harman, a standard solution of 1S,3S-MTCA (Sigma) 50 μM in 100 mM phosphate buffer pH 4 was heated (80 °C, 3 h) or added to H_2_O_2_ (2 mM) (1 h), NaNO_2_ (100 μM) (1 h), or H_2_O_2_ (50 μM) plus FeSO4 (50 μM) (1 h), and subsequently analyzed by RP-HPLC for harman as indicated below. Also, the PRS extracts isolated from soy sauce were successively injected into the RP-HPLC as mentioned below, and the peaks corresponding to 1*S*,3*S*-MTCA and 1*R*,3*S*-MTCA collected and heated at 90 °C for 3–6 h and subsequently analyzed for harman as indicated below. On the other hand, a sample of soy sauce was treated under several conditions: control (37 °C, 2 h), heating (90 °C, 2 h), H_2_O_2_ (5 mM) or FeSO_4_ (5 mM) plus H_2_O_2_ (5 mM) (37 °C, 2 h), and subsequently analyzed for harman.

### 4.3. Monoamine Oxidase (MAO-A and B) Assay and Inhibition by Soy Sauce and β-Carbolines Isolated from Soy Sauce

A chromatographic assay was carried out to determine MAO activity [17,21,50]. It was performed with membrane protein fractions containing MAO-A or MAO-B that were diluted to the desired concentrations in 100 mM potassium phosphate buffer (pH 7.4) [21,50]. A 0.2 mL reaction mixture containing 0.01 mg/mL protein and 0.25 mM kynuramine in 100 mM potassium phosphate (pH 7.4) was incubated at 37 °C for 40 min. After incubation, the reaction was stopped by the addition of 2N NaOH (75 μL), followed by the addition of 70% HClO_4_ (25 μL), and the sample was centrifuged (10,000× *g*) for 5 min. Under these conditions, kynuramine deaminated by MAO spontaneously cyclizes to give 4-hydroxyquinoline. An aliquot of the supernatant (20 μL) was injected into the HPLC, and the deamination product of kynuramine (i.e., 4-hydroxyquinoline) formed during the enzymatic reaction determined by RP-HPLC-DAD at 320 nm. A response curve of area versus concentration was constructed to calculate the concentration of 4-hydroxyquinoline. The standard inhibitors of MAO, clorgyline (MAO-A inhibitor) and deprenyl (MAO-B inhibitor) were used as reference inhibitors in the assays (1 µM).

The inhibition assays were performed with: (a) aliquots of diluted soy sauce (diluted 1/3 in 100 mM phosphate buffer pH 7.4 with 10% of DMSO), (b) soy sauce extracts and fractions prepared from SPE containing the β-carbolines (i.e., buffer-methanol PRS fractions), and (c) samples of isolated harman from soy sauces. For that, aliquots of those samples were added to reaction mixtures containing kynuramine (0.25 mM) and MAO enzyme (A or -B; 0.01 mg/mL membrane protein) in 100 mM potassium phosphate buffer (pH 7.4), as above. Corresponding blanks and controls were used. The MAO kinetic and the mechanism of inhibition were assessed by analyzing the corresponding Michaelis-Menten curves and double reciprocal Lineweaver-Burk plots obtained at different concentrations of the substrate kynuramine. To determine MAO-binding reversibility, membrane proteins of MAO-A and B (0.12 mg/mL) in 100 mM phosphate buffer (pH 7.4) were preincubated (37 °C, 40 min) with diluted soy sauce aliquots. The mixtures were centrifuged (15,000× *g*) for 15 min to pellet membrane proteins, washed twice with 100 mM phosphate buffer, and finally, the pellet was resuspended in 100 mM phosphate buffer + 10% DMSO (0.1 mL). An aliquot (40 μL) was used to measure MAO activity and compared with corresponding controls. 

### 4.4. RP-HPLC chromatographic Analysis and Chemical Identification by MS

The analysis of 4-hydroxyquinoline (kynuramine deamination product), β-carbolines and tetrahydro-β-carbolines was performed by RP-HPLC with *uv-DAD* and fluorescence detection using an HPLC 1050 (Agilent Technologies, Santa Clara, CA, USA) with a Diode Array Detector (DAD) and a 1046A-fluorescence detector. A 150 mm × 3.9 mm, 4 μm, Nova-pak C18 column (Waters, Milford, MA, USA) was used for chromatographic separation. Chromatographic conditions were 50 mM ammonium phosphate buffer (pH 3) (buffer A) and 20% of A in acetonitrile (buffer B). Gradient programmed from 0% (100% A) to 32% B in 8 min, and 90% B in 15 min. The flow rate was 1 mL/min, the column temperature was 40 °C, and the injection volume was 20 μL. Absorbance detection was set at 320 nm (analysis of 4-hydroxyquinoline), whereas fluorescence detection was used for tetrahydro-β-carbolines (270 nm, excitation and 343 nm, emission) and norharman and harman (300 nm, excitation and 433 nm, emission). Identification of compounds was done by UV, fluorescence and mass spectrometry. The identification by HPLC-ESI-mass spectrometry of 4-hydroxyquinoline in MAO assays was carried out as previously [50]. Identification of the β-carbolines norharman and harman in soy sauce was carried out in the PRS extracts, and the THβC-3-COOH in SCX extracts was obtained as mentioned above. They were analyzed with a 2.1 × 150 mm Zorbax SB-C18, 5 μm, column (Agilent Technologies, Santa Clara, CA, USA) by using an HPLC-MSD series 1100 (Agilent) (electrospray-positive ion mode). Eluent A: formic acid (0.5%); B: formic acid (0.5%) in acetonitrile; 80% B in 30 min, flow rate 0.25 mL/min.; T: 40 °C; mass range 50–700 u and cone voltage 100 V.

## 5. Conclusions

This work has shown that soy sauce inhibits MAO-A and -B isozymes in vitro, as evidenced by an assay of kynuramine deamination performed by chromatographic means, suggesting the occurrence of MAO inhibitors in soy sauces. The inhibition of MAO was reversible for MAO-A and partly reversible for MAO-B. Soy sauces were analyzed, and the β-carboline alkaloids, norharman and harman, were identified. Subsequently, harman was isolated from soy sauce, and it potently inhibited MAO-A in a competitive mode. This β-carboline was analyzed in several soy sauces, and it reached a concentration of 243 μg/L. In addition, the THβC-3-COOH compounds, THCA and MTCA, were identified and analyzed in soy sauces. MTCA isolated from soy sauce afforded harman under conditions of heating and oxidation. Also, under heating and oxidative conditions, soy sauces increased the levels of harman, showing that MTCA was the precursor of harman in soy sauces. Then, soy sauce contained β-carboline alkaloids that are considered bioactive compounds, and among them, harman is a potent inhibitor of MAO-A, as shown here. Results suggest that soy sauce constitutes a dietary source of MAO inhibitors.

## Figures and Tables

**Table 1:** Concentration of aromatic β-carbolines and THβC-3-COOH alkaloids in different commercial soy sauces (*n* = 7).

β-Carbolines	X (μg/L)	Range
Norharman	34.9	11.6–52
Harman	165.4	46.7–243
**THβC-3-COOH**	**X (mg/L)**	**Range**
THCA	18.8	2.2–69.6
1*S*,3*S*-MTCA	161.6	43.6–360.5
1*R*,3*S*-MTCA	42.8	8.9–88.2

## Data Availability

The data are included in this article.

## Sample Availability

Samples of the compounds are available from the authors.
